# Aspect Ratio Model for Radiation-Tolerant Dummy Gate-Assisted n-MOSFET Layout

**DOI:** 10.1155/2014/145759

**Published:** 2014-11-17

**Authors:** Min Su Lee, Hee Chul Lee

**Affiliations:** Division of Electrical Engineering, School of Electrical Engineering & Computer Science, Korea Advanced Institute of Science and Technology, 373-1 Guseong-dong, Yuseong-gu, Daejeon 305-701, Republic of Korea

## Abstract

In order to acquire radiation-tolerant characteristics in integrated circuits, a dummy gate-assisted n-type metal oxide semiconductor field effect transistor (DGA n-MOSFET) layout was adopted. The DGA n-MOSFET has a different channel shape compared with the standard n-MOSFET. The standard n-MOSFET has a rectangular channel shape, whereas the DGA n-MOSFET has an extended rectangular shape at the edge of the source and drain, which affects its aspect ratio. In order to increase its practical use, a new aspect ratio model is proposed for the DGA n-MOSFET and this model is evaluated through three-dimensional simulations and measurements of the fabricated devices. The proposed aspect ratio model for the DGA n-MOSFET exhibits good agreement with the simulation and measurement results.

## 1. Introduction

**Figure 1:**
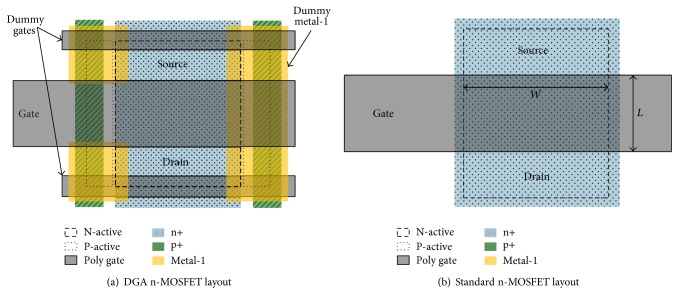
(a) DGA n-MOSFET layout and (b) standard n-MOSFET layout.

According to a recent report [1], the dummy gate-assisted n-type metal oxide semiconductor field effect transistor (DGA n-MOSFET) layout, as shown in Figure 1(a), has radiation-tolerant characteristics with regard to a total ionizing dose up to 500 krad (Si). The DGA n-MOSFET layout eliminated all possible radiation-induced leakage currents through the isolation of the source and drain from the sidewall oxides using dummy gates and P-active layers involving p+ layers. In the DGA n-MOSFET, the isolation of the source and drain from the sidewall oxides results in a different channel shape compared with that of the standard n-MOSFET.

The standard n-MOSFET, which is shown in Figure 1(b), has a channel region with a rectangular shape, which has a width *W* and length *L*. Furthermore, this simple geometry enables easy calculation of its aspect ratio. The current density of the standard n-MOSFET is constant along its length [2]. Therefore, the aspect ratio of the standard n-MOSFET is calculated through dividing *W* by *L*. When the integrated circuit is designed using standard MOSFETs, the circuit characteristics are determined using adjustments of each aspect ratio of *W*/*L* in the MOSFETs. Circuit designers can adjust only the aspect ratios in order to obtain specified circuit characteristics because the other parameters are determined in the device fabrication process. Therefore, the aspect ratio of the MOSFET is the most important parameter in the circuit design.

**Figure 2:**
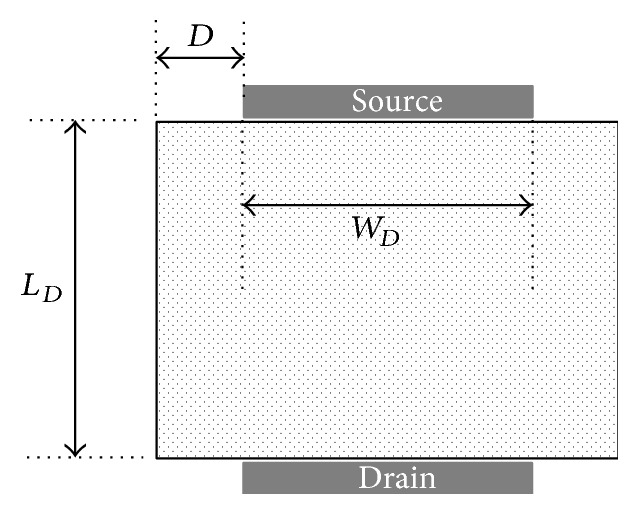
The channel region in the DGA n-MOSFET layout. The channel of the DGA n-MOSFET layout has an extended channel area at the edge of the source and drain compared with the standard n-MOSFET. *L*_*D*_, *W*_*D*_, and *D* are the channel length, channel width, and extended width, respectively.

The channel shape of the DGA n-MOSFET is presented in Figure 2 with the removal of the areas other than the channel area. Its current density is not constant along its length due to its inherent structure shape. Therefore, the aspect ratio of the DGA n-MOSFET cannot be obtained in the same manner as a standard n-MOSFET. As a result, a specified aspect ratio model for the DGA n-MOSFET should be developed.

According to previous reports [3, 4], the resistance, which has a shape similar to the channel region of the DGA n-MOSFET, does not have an analytical solution. Therefore, several alternative formulas have been reported and evaluated through the adoption of approximations. However, these formulas have very high complexity and difficulty to be adopted for the DGA n-MOSFET. Furthermore, the specified aspect ratio model for the DGA n-MOSFET has not been developed yet.

For the practical use of the DGA n-MOSFET in circuit design, this study proposes a simple aspect ratio model by adopting several reasonable approximations. It is expected that the proposed simple aspect ratio model simplifies the estimation of the aspect ratio of the DGA n-MOSFET. The proposed aspect ratio model is evaluated using a three-dimensional simulation with the SILVACO ATLAS simulation tool. Furthermore, the proposed aspect ratio model is also evaluated through device fabrication and its measurement.

## 2. Proposed Aspect Ratio Model

### 2.1. Aspect Ratio Model for the Trapezoidal Structure

**Figure 3:**
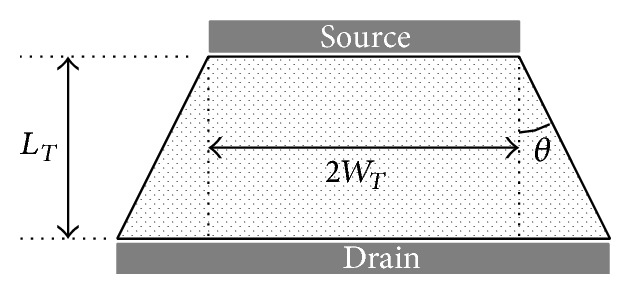
The transistor with a trapezoidal channel shape. *L*_*T*_, *W*_*T*_, and  *θ* are the channel length, half of the source side channel width, and corner angle, respectively.

Figure 3 shows the trapezoidal structure with its parameters. The aspect ratio of the trapezoidal structure was modeled from an approximated triode region drain current based on the gradual channel approximation and charge sheet assumption [2]. The effective aspect ratio of the trapezoidal structure can be calculated using the following equation:

(1) $$\begin{array}{c} {\left(\frac{W}{L}\right)}_{\text{eff}}^{\text{Trap}}=\frac{2\theta }{\ln\left(\left(W_{T}+\theta L_{T}\right)/W_{T}\right)}, \end{array}$$

where *L*_*T*_, *W*_*T*_, and  *θ* are the channel length, half of the source side channel width, and corner angle, respectively [2].

### 2.2. Aspect Ratio Model for the DGA n-MOSFET

The channel region of the DGA n-MOSFET is approximated using the series association of two trapezoidal transistors. Moreover, the value of the corner angle *θ* was specified in order to determine whether to place an additional rectangle between the two trapezoid transistors. Thus, it was determined that if (*L*_*D*_ · tan⁡*θ*)/2 > *D*, a rectangle is added between the two trapezoid transistors. *L*_*D*_, *D*, and *θ* are the channel length, extended width, and corner angle, respectively. This approximation was developed based on two assumptions. The first assumption is that the current density at the corner regions of the DGA n-MOSFET channel can be negligible. It is intuitively known that the electric field will not reach the corner regions; consequently, the series association of the two trapezoidal structures substitutes the DGA n-MOSFET channel region. The second assumption is that the corner angle *θ* of the substituted trapezoid structure will be constant regardless of the length *L*_*D*_, extended width *D*, and width *W*_*D*_, because the spreading angle of the current at the edge of the source is expected to be constant. The behavior of the current spreading is determined by the contact shape between the electrodes and channel region. In the DGA n-MOSFET, the source and drain edges are always positioned in the middle of the upper and lower sides of the channel region.

**Figure 4:**
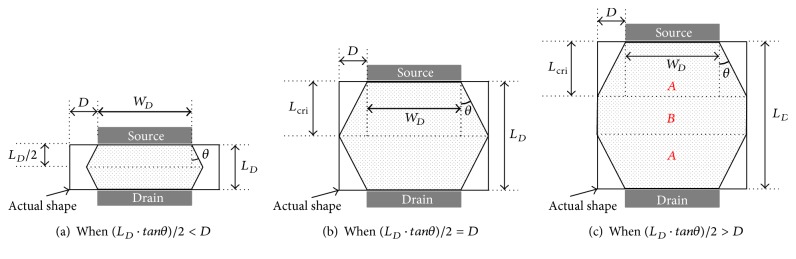
Approximated channel shapes of the DGA n-MOSFET: (a) when (*L*_*D*_ · tan⁡*θ*)/2 < *D*, (b) when (*L*_*D*_ · tan⁡*θ*)/2 = *D*, and (c) when (*L*_*D*_ · tan⁡*θ*)/2 > *D*. *L*_*D*_, *W*_*D*_, *D*, and *θ* are the channel length, channel width, extended width, and corner angle, respectively.

The final approximated channel shapes are presented in Figure 4. In Figures 4(a) to 4(c), the width *W*_*D*_, extended width *D*, and corner angle *θ* have the same values; however, the length *L*_*D*_ has different values and it increases in order of the figure number.  Figure 4(a) presents the case with the condition of (*L*_*D*_ · tan⁡*θ*)/2 < *D*.

In this case, the series association of the two trapezoidal transistors replaces the channel area of the DGA n-MOSFET. Moreover, a space exists between the bottom vertexes of the upper trapezoid and both left and right sides of the original channel area. The effective aspect ratio for the approximated structure shown in Figure 4(a) is calculated using the following equation:

(2) $$\begin{array}{c} {\left(\frac{W}{L}\right)}_{\text{eff}}^{\text{DGA}\_(\text{a})}=\frac{\theta }{\ln\left(\left(W_{D}+\theta L_{D}\right)/W_{D}\right)}, \end{array}$$

where *L*_*D*_, *W*_*D*_, and  *θ* are the channel length, channel width, and corner angle, respectively.

Figure 4(b) represents the case when the condition is (*L*_*D*_ · tan⁡*θ*)/2 = *D*. In this case, the series association of the two trapezoidal transistors also replaces the channel area of the DGA n-MOSFET. However, the bottom vertexes of the upper trapezoid encounter both left and right sides of the original channel area. The effective aspect ratio for the approximated structure shown in Figure 4(b) is calculated using the following equation:

(3) $$\begin{array}{c} {\left(\frac{W}{L}\right)}_{\text{eff}}^{\text{DGA}\_(\text{b})}=\frac{\theta }{\ln\left(\left(W_{D}+\theta L_{D}\right)/W_{D}\right)}, \end{array}$$

where *L*_*D*_( = 2*L*_cri_), *W*_*D*_, and  *θ* are the channel length, channel width, and corner angle, respectively.

Figure 4(c) represents the case when the condition is (*L*_*D*_ · tan⁡*θ*)/2 > *D*. In this case, the series association of the two trapezoidal transistors and one rectangle replaces the channel area of the DGA n-MOSFET. The rectangle is added between the two trapezoidal structures. The effective aspect ratio for the approximated structure shown in Figure 4(c) is calculated using the following equations:

(4) $$\begin{array}{c} {\left(\frac{W}{L}\right)}_{\text{eff}}^{\text{DGA}\_(\text{c})}=\frac{1}{2/A+1/B},\\ A=\frac{2\theta }{\ln\left(\left(W_{D}+2\theta L_{\text{cri}}\right)/W_{D}\right)},\;\;B=\frac{W_{D}+2D}{L_{D}-2L_{\text{cri}}},\\ L_{\text{cri}}=\frac{D}{\tan\theta }, \end{array}$$

where *L*_*D*_, *W*_*D*_, *D*, and  *θ* are the channel length, channel width, extended width, and corner angle, respectively. Moreover, *A* and *B* indicate the aspect ratio formula for the trapezoidal structure and rectangle, respectively, as shown in Figure 4(c).

## 3. Simulation Results

### 3.1. Evaluation of Current Density

The standard n-MOSFET structure and DGA n-MOSFET structure were designed to be compatible with the commercial 0.35 *μ*m process technology using the SILVACO ATLAS simulation tool. For both the standard and DGA n-MOSFETs, the gate oxide thickness, body thickness, and body doping density were 7 nm, 6 *μ*m, and 8e16 #/cm^3^, respectively. For the DGA n-MOSFET, the side p+ doping density was 1e19 #/cm^3^. Moreover, the isolation structure was adapted using local oxidation of silicon (LOCOS). Five different widths (2 *μ*m, 5 *μ*m, 8 *μ*m, 10 *μ*m, and 12 *μ*m) and three different lengths (0.5 *μ*m, 1 *μ*m, and 3 *μ*m), which resulted in fifteen different structures, were simulated. For the DGA n-MOSFET, the extended width *D* was fixed to 2 *μ*m.

**Figure 5:**
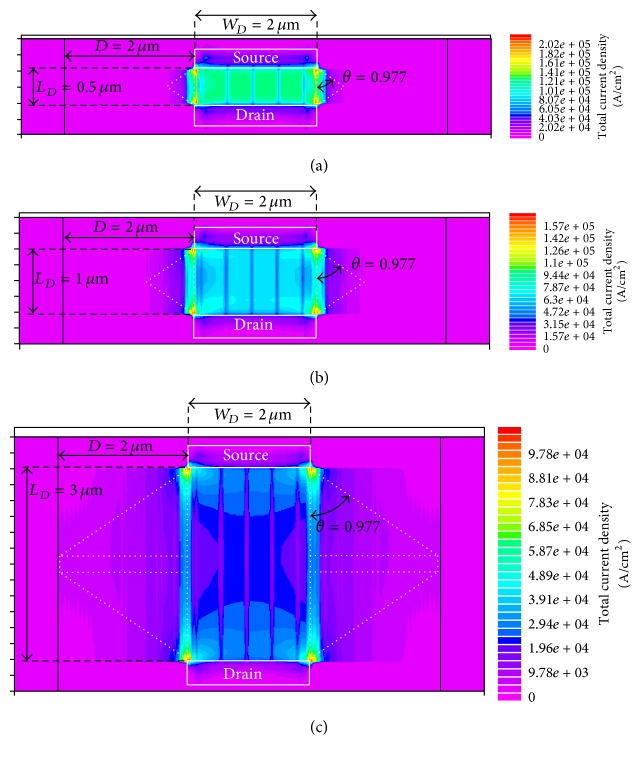
Simulated current density of the DGA n-MOSFETs at the channel surface when the gate, source, drain, and body voltages are 3.3 V, 0 V, 0.05 V, and 0 V, respectively. Current densities are presented using rainbow colors relative to its maximum current density. All *W*_*D*_ and *D* are 2 *μ*m; *L*_*D*_ increases in order of the figure number as follows: (a) 0.5 *μ*m, (b) 1 *μ*m, and (c) 3 *μ*m. The white dashed lines indicate the approximated channel area when  *θ* is 0.977.

Figure 5 presents the current density of the DGA n-MOSFETs at the channel surface when the gate, source, drain, and body voltages were 3.3 V, 0 V, 0.05 V, and 0 V, respectively. In Figures 5(a) to 5(c), both the width *W*_*D*_ and extended width *D* were 2 *μ*m. The length *L*_*D*_ increased in order of the figure number as 0.5 *μ*m, 1 *μ*m, and 3 *μ*m. In order to evaluate the proposed effective aspect ratio model for the DGA n-MOSFET, the current densities are presented using rainbow colors in a linear scale relative to each maximum current density. From Figure 5, it can be seen that the currents increasingly expanded to the extended channel area as the channel length increased.

The corner angle  *θ* was determined using comparisons of the proposed effective aspect ratio model with the simulation results. When the corner angle  *θ* was 0.977, the proposed effective aspect ratio model had the best agreement with the simulation results. The approximated channel area with a corner angle  *θ* of 0.977 is indicated in Figure 5 using the white dashed lines. These white dashed lines also agree well with the current density profile.

### 3.2. Extracted Effective Aspect Ratio of the DGA n-MOSFET

In the simulation, the effective aspect ratios for the DGA n-MOSFETs were extracted using the following equations:

(5) $$\begin{array}{c} g_{m}=\frac{\partial I_{d}}{\partial V_{gs}}=\frac{W_{\text{eff}}}{L_{\text{eff}}}\mu_{n}C_{\text{ox}}V_{ds}\left(1+\lambda V_{ds}\right),\\ W_{\text{eff}}=W_{\text{drawn}}-\Delta W,\\ L_{\text{eff}}=L_{\text{drawn}}-\Delta L,\\ {\left(\frac{W_{\text{eff}}}{L_{\text{eff}}}\right)}_{\text{eff}}^{\text{DGA}}={\left(\frac{W_{\text{eff}}}{L_{\text{eff}}}\right)}_{\text{eff}}^{\text{STD}}\frac{{g_{m}}^{\text{DGA}}}{{g_{m}}^{\text{STD}}}, \end{array}$$

where *W*_drawn_,  *W*_eff_,  *L*_drawn_,  *L*_eff_, *μ*_*n*_, *C*_ox_, and  *λ* are the drawn width, effective width, drawn length, effective length, electron mobility, gate oxide capacitance per unit area, and channel length modulation parameter, respectively. Moreover, the superscripts “DGA” and “STD” refer to the DGA n-MOSFET and standard n-MOSFET, respectively. When calculating the effective aspect ratios, it was assumed that the DGA n-MOSFET and standard n-MOSFET have the same electrical parameters for the *μ*_*n*_ *C*_ox_ and channel length modulation parameter  *λ*. The transconductance *g*_*m*_ was adopted at a *V*_*ds*_ of 0.05 V in order to minimize the effects induced by the channel length modulation parameter  *λ* difference between the DGA n-MOSFET and standard n-MOSFET. Furthermore, the transconductance *g*_*m*_ was used at a *V*_*gs*_ of 1.5 V in order to use a saturated *g*_*m*_.

Moreover, in order to acquire the effective width *W*_eff_ and effective length *L*_eff_, the Δ*W* and Δ*L* were extracted from the simulation results of the standard n-MOSFETs. From the simulation results, the extracted Δ*W* and Δ*L* were 0.05 *μ*m and 0.005 *μ*m, respectively.

**Figure 6:**
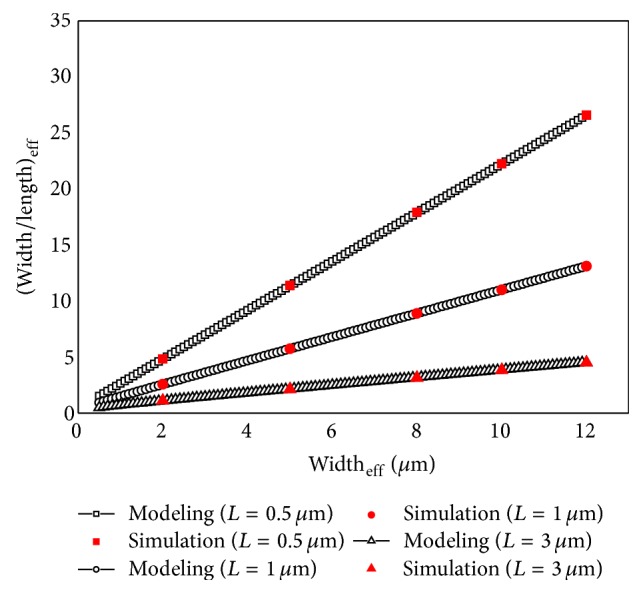
Comparison of the effective aspect ratios of the DGA n-MOSFETs when calculated using the proposed effective aspect ratio model and when extracted from the three-dimensional simulation.

Figure 6 shows the calculated aspect ratios using the proposed aspect ratio model and the extracted aspect ratios from the simulation results. The errors of the proposed effective aspect ratio model compared with those of the simulation results were contained between −0.4% and 1.4%. The proposed effective aspect ratio model was well matched with the simulation results.

## 4. Experimental Results

The standard n-MOSFET and DGA n-MOSFET were fabricated using the MagnaChip and Hynix 0.35 *μ*m technology with a gate oxide thickness of 7.3 nm and LOCOS for the isolation of the active areas. Three different widths (5 *μ*m, 8 *μ*m, and 12 *μ*m) and three different lengths (0.5 *μ*m, 1 *μ*m, and 3 *μ*m), which resulted in a total of nine structures, were fabricated. For the DGA n-MOSFET, the extended side channel width *D* was fixed to 2 *μ*m.

The fabricated devices were measured using an Agilent 4156A semiconductor parameter analyzer. The *V*_*g*_ *-I*_*d*_ curves were measured and were then differentiated by *V*_*g*_ in order to acquire the transconductance *g*_*m*_ curve. When measuring the *V*_*g*_ *-I*_*d*_ curves, the *V*_*ds*_ was set to 0.05 V. Moreover, all transconductance values *g*_*m*_ used to extract the aspect ratios were chosen at a *V*_*gs*_ of 1.5 V.

As in the simulation case, in order to extract the effective aspect ratios for the DGA n-MOSFETs, (5) were also adopted. Moreover, from the measurement results, the extracted Δ*W* and Δ*L* were 0.15 *μ*m and 0.05 *μ*m, respectively.

**Figure 7:**
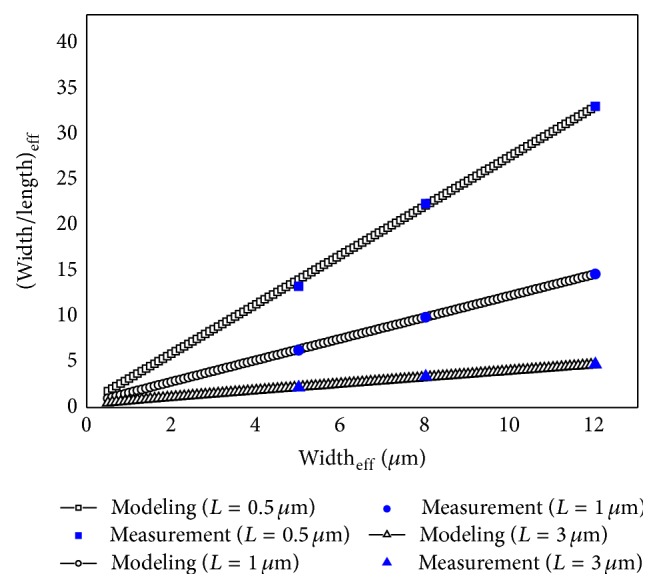
Comparison of the effective aspect ratios of the DGA n-MOSFETs when calculated using the proposed effective aspect ratio model and when extracted from the measurement.

Figure 7 shows the calculated aspect ratios using the proposed aspect ratio model and the extracted aspect ratios from the measurement results. The errors of the proposed effective aspect ratio model compared with the measurement results were contained between −3% and 5.4%. When considering the process variation, which was ±5%, the error range is acceptable. Therefore, the proposed effective aspect ratio model was well matched with the measurement results.

## 5. Conclusion

For practical use of the radiation-tolerant DGA n-MOSFET in designing a radiation-tolerant integrated circuit, an effective aspect ratio model of the DGA n-MOSFET was proposed. In the proposed effective aspect ratio model, the channel area of the DGA n-MOSFET was approximated using a trapezoidal structure and a rectangular structure. When (*L*_*D*_ · tan⁡*θ*)/2 > *D*, the rectangular structure was added between the two trapezoidal structures.

The proposed effective aspect ratio model was evaluated using a three-dimensional simulation and through measurement of the fabricated devices. Compared with the simulation results, the proposed effective aspect ratio model had an error range between −0.4% and 1.4%. Moreover, compared with the measurement results, the proposed effective aspect ratio model had an error range between −3% and 5.4%. Therefore, it can be concluded that the proposed effective aspect ratio model is well matched with the simulation and measurement results.
